# Final-Year Dental Students’ Perceived Confidence: Competencies in General Dentistry

**DOI:** 10.3390/dj13060268

**Published:** 2025-06-16

**Authors:** Navodika Yaparathna, Iresha Udayamalee, Megan Gray, Cheree He, Rachel Wu, Chelsea Taing, Menaka Abuzar

**Affiliations:** School of Medicine and Dentistry, Griffith University, Southport, QLD 4215, Australia; s.udayamalee@griffith.edu.au (I.U.); megan.gray@griffith.edu.au (M.G.); cheree.he@griffithuni.edu.au (C.H.); rachel.wu@griffithuni.edu.au (R.W.); chelsea.taing@griffithuni.edu.au (C.T.)

**Keywords:** professional competence, dentistry, outplacement training, final-year dental students, confidence, dental curriculum

## Abstract

**Background:** Competency in providing high-quality, comprehensive patient care is essential for newly qualified dentists. Dental curricula are designed to equip graduates with necessary skills to develop competencies. Outplacement training has been incorporated into most dental curricula to provide broader clinical experience in a real-world situation. **Methods:** This cross-sectional study aimed to assess (1) the perceived confidence of final-year dental students (FYDSs) at an Australian university with reference to the Australian Dental Council (ADC) professional competencies for newly qualified dentists; (2) the association between perceived confidence and the timing of outplacement training; and (3) students’ perceptions on outplacement training in developing their competencies. Two online surveys were administered to a cohort of FYDSs at the end of the first and second trimesters. ‘Survey 1’ was based on the ADC competency requirements (2022) and assessed aims 1 and 2. The five domains assessed were (1) communication and leadership; (2) critical thinking; (3) health promotion; (4) scientific and clinical knowledge; and (5) person-centred care. ‘Survey 2’ assessed students’ perception on outplacement training and was administered towards the completion of their outplacement to assess aim 3. **Results:** Students’ perceived competency levels were high across all domains. Those with prior tertiary education were more confident in communication and leadership, health promotion, and scientific and clinical knowledge than students with secondary qualifications. The perceived confidence in professional competencies among FYDSs had no significant association (*p* > 0.05) with the location or the sequence of outplacement. The thematic analysis of survey 2 responses reported the guidance and constructive feedback received from supervisors while managing cases in a real-life setup as a significant contributor to their confidence development. **Conclusions:** FYDSs reported a satisfactory level of perceived confidence in professional competencies. Both in-house training and outplacement equally improve the levels of competency development. FYDSs perceive outplacement training as a positive integral component in the development of skills.

## 1. Introduction

Competency in the provision of high-quality, comprehensive patient care is essential for newly qualified dentists. Therefore, dental curricula should be designed and routinely refined to align with current standards to provide students with the skills and knowledge needed for a seamless transition into general dental practice [1]. Dental regulatory bodies are official organisations established in various countries to regulate the practice of dentistry and related fields, ensuring that dental professionals adhere to high standards of care, ethics, and competency. These bodies typically operate under governmental or legal authority and play a critical role in public health by overseeing dental education, licencing, and professional conduct. The Australian Dental Council (ADC) is the regulatory body in Australia.

Competency is the ability to combine evidence-based knowledge and undertake independent and holistic dental practices [2]. These competencies are developed during their education at dental school through curriculum content and practical training in university clinics and at outplacements. This allows students to gain diverse clinical experiences and foster independence [3,4]. Reflective practice of these skills is essential to evaluate preparedness in providing safe clinical care after graduation [1]. However, newly graduated dentists often feel underprepared due to insufficient clinical exposure, highlighting the need for curriculum adjustments to extend hands-on training [3].

The ADC publication ‘Professional competencies of the newly qualified dental practitioner’ has been developed using resources and recommendations from international dental accreditation organisations [5]. This document outlines the competencies required for newly qualified dental practitioners to be eligible for registration in Australia. It also states the accreditation standards for Australian dental education programmes. The expected competencies for newly qualified dentists are clustered into six key competency domains: 1. social responsibility and professionalism (SRP); 2. communication and leadership (CL); 3. critical thinking (CT); 4. health promotion (HP); 5. scientific and clinical knowledge (SCK); and 6. person-centred care (PCC) (The Australian Dental Council, 2022).

There is a paucity of research assessing the perceived competency of FYDSs worldwide [3,6,7]. The available literature is also limited to studies focusing on specific areas such as clinical skills [8,9], cultural competency [10], and practice management [11]. The previous studies have reported the high levels of perceived confidence in common procedures such as history taking, simple operative and preventative treatments. However, the confidence levels reported were very low in advanced procedures such as surgical extractions involving flaps and molar endodontics [6,12]. The only available Australian study assessing the perceived competency levels of FYDSs was based on the 2010 published ADC guidelines [12]. The study revealed an overestimation of self-confidence among Australian FYDSs and emphasised the need for a deeper understanding of how current dental training aligns with the competencies outlined by the ADC.

Most Australian dental schools provide students clinical training at university clinics and at outplacement clinics. Outplacement training has become an important component of the dental curriculum as it provides students clinical experience in a ‘real-world’ situation during the pre-graduation year. It is undertaken away from the university hospital, usually in government clinics situated in metropolitan, regional, and/or rural locations and has proven to benefit students’ confidence, competence, and professionalism through its diverse clinical experience [13].

Understanding students’ perception of expected competency levels on completion of their studies assist institutions to structure their programmes to improve dental students’ transition into professional practice [14]. Therefore, the aims of this study were to investigate the perceived confidence of FYDSs at an Australian university using an online survey based on the 2022 ADC professional competencies for newly qualified dentists (with respect to five domains: CL, CT, HP, SCK, and PCC), the association between perceived confidence and the timing of outplacement training, and the students’ perceptions on outplacement training in confidence development.

## 2. Materials and Methods

A cross-sectional research design was employed to evaluate FYDSs’ perceived confidence levels in 5 of the competencies stated by the ADC. The association between perceived confidence and outplacement training in developing competencies was also investigated qualitatively and quantitatively. The sample population was FYDSs enrolled in the final year with the sampling frame obtained from the official enrolment list 2023. Partially completed responses were excluded. Ethical approval was gained from the University Human Research Ethics Committee (GU Ref No: 2023/378).

### 2.1. Background and Clinical Training Schedule

The university academic year is conducted as a trimester system with three study periods per academic year. Trimester 1 (T1) is the first half from January to June, trimester 2 (T2) is the second half from July to end of October of the academic year, and the third is a summer trimester (November and December). Dentistry clinical training occurs in T1 and T2. The final-year cohort is divided into two groups to accommodate students in outplacement clinics and in-house (university clinic). One group undertakes in-house training at the university dental clinic, while the second group completes outplacement training in various regional and metropolitan public dental clinics over approximately 17 weeks during T1. In T2, the groups alternate training locations, with those students that were in outplacements in T1 coming back to the university clinic.

### 2.2. Survey 1: Data Collection and Analysis

In order to assess the objectives of this study, two questionnaires (Questionnaire 1: Appendix A; Questionnaire 2: Appendix B) were developed and administered as online surveys. Survey 1 was administered twice to the entire cohort of FYDSs (‘Survey 1 T1′ at the end of T1 and ‘Survey 1 T2′ at the end of T2) through LimeSurvey, an approved third-party online research survey tool platform [15]. Request for participation was sent via email to the entire cohort. When conducting the survey, informed consent was obtained from all participants by providing a plain language statement (PLS), comprehensive information about the study, and a link to the questionnaire. This prevented potential identifying factors from being linked with their responses [16]. Participation was voluntary, and no conflicts were identified.

Questionnaire 1 (Appendix A) was based on the document ‘Professional competencies of the newly qualified dental practitioner,’ ADC, 2022, to assess self-reported confidence. Questionnaire 1 consisted of two sections: (1) demographic data and (2) statements to assess the professional competencies of newly qualified dental practitioners extracted from the 2022 ADC document. Demographic characteristics included age, gender, outplacement trimester, and completion of any previous tertiary education. Professional competencies included 51 assessment outcomes under the 5 domains selected for this study: (1) CL (9 statements); (2) CT (3 statements); (3) HP (4 statements); (4) SCK (7 statements); and (5) PCC (28 statements). The domain social responsibility and professionalism (SRP), which includes 12 statements, was excluded from this questionnaire and will be assessed in detail in a separate study. Respondents rated their level of perceived confidence on a 10-point linear numeric scale from 1: ‘No confidence’ to 10: ‘Very confident’. The CL domain evaluates the ability to collaborate and communicate professionally; the CT domain focuses on the acquisition and application of knowledge; the HP domain covers community health promotion; the SCK domain assesses the application of essential knowledge required by dental practitioners; and the PCC domain evaluates clinical information gathering, diagnosis, and clinical treatment.

Data cleaning was performed, and incomplete responses were removed. The data was analysed using descriptive and inferential statistics via IBM SPSS Statistics Version 29. The perceived confidence levels for each domain (CL, CT, HP, SCK, and PCC) were evaluated by developing composite values using the scores for the questions. Overall confidence was calculated by the cumulative value of all the domains, assessed with all 51 questions. A median value of eight was used as the central tendency to determine the confidence level for each domain, as a perfect Gaussian distribution was not present. Those who obtained a higher value than the central tendency level were considered to have “more than median level of confidence” and their counterparts were considered to have “less than median level of confidence”. Descriptive statistics were used to assess frequencies, central tendency values, and frequency distributions. Comparisons between the numeric scores from the in-house and outplacement groups were performed using non-parametric chi-square tests and Fisher’s exact tests. Any *p*-values less than 0.05 were considered statistically significant.

### 2.3. Survey 2: Data Collection and Analysis

Questionnaire 2 was developed for a qualitative and quantitative assessment of students’ perception of their outplacement training. The face and content validity of the questionnaire was verified with an expert team of dental educators and distributed as an online survey (Survey 2). The student group that completed outplacement training in T1 completed the survey in June, while the other group (T2) completed it in November. The data was collected maintaining anonymity. The questionnaire consisted of 10 open-ended questions and 6 questions to rate their perception of the learning experience during outplacement training on a 10-point linear numeric scale.

Qualitative data was analysed using basic natural language processing (NLP) techniques in Python 3.12.x (2023) programming language. Thematic analysis was conducted using recurring patterns and key themes given by the students in their qualitative responses. The responses were categorised into predefined groups with content analysis based on their relevance. Sentiment analysis was conducted using TextBlob Python NLP library [17] and assigned a polarity score ranging from −1 to + 1. This classified the responses as positive, neutral, or negative. The data was quantitatively analysed using descriptive and inferential statistics via IBM SPSS Statistics Version 29.

## 3. Results

### 3.1. Survey 1

A total of 68 out of 91 FYDSs responded to Survey 1 T1, representing a response rate of 75.8%, while 35 out of 91 FYDSs responded to Survey 1 T2, representing a response rate of 38.5%. Their sociodemographic characteristics are described in Table 1.

**Figure 1:**
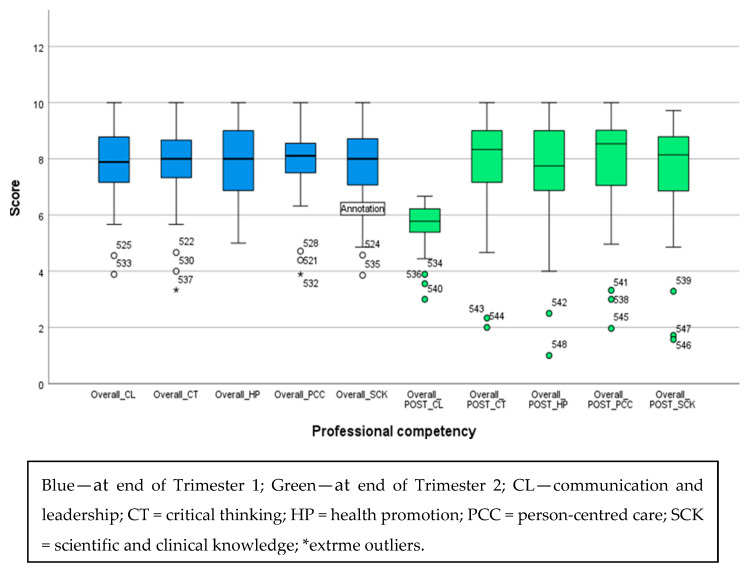
Box plot representation of final-year dental students’ perceived confidence at the end of T1 and T2.

At the end of T1 and T2, FYDSs demonstrated a higher level of overall confidence in all the domains (Figure 1). At the end of T1, the perceived confidence levels for CL, CT, HP, SCK, and PCC were 7.88, 8.00, 8.00, 8.00, and 8.10, respectively. These values represent the median confidence level for each domain. The overall confidence level was 8.05 with a Standard Deviation (SD) ± 1.06. At the end of T2, it was interesting to note that the confidence level for CL had decreased to 5.77, while CT, HP, SCK, and PCC remained consistent at 8.33, 7.75, 8.14, and 8.53, respectively. The cumulative overall confidence at the end of T2 was 8.18 (SD ± 1.834). The skewness was −1.654 and the kurtosis was 0.398. The left skew indicated that more participants had higher confidence scores, with a few lower outliers pulling the tail to the left. Despite having fewer outliers, the majority reported a higher level of confidence of around eight, as shown in Figure 1.

Out of 42 students who completed outplacement in T1, 23 (54.7%) reported an overall confidence level above the cohort’s median of eight. However, when considering the midpoint of the scale (5), 95.2% (*n* = 40) of students perceived their confidence as above mid-level. In relation to in-house training in T1, 14 out of 26 students (53.8%) reported an overall confidence level above the cohort’s median of eight. When considering the midpoint of the scale (5), 100% (*n* = 26) of students perceived their confidence as above mid-level.

Of the 20 students who completed outplacement training in T2, 12 students (60%) reported an overall confidence level above the cohort’s median of eight. When considering the midpoint of the scale (5), 85% (*n* = 17) of students perceived their confidence as being above the mid-level. Regarding in-house training in T2, 9 out of 15 students (60%) reported an overall confidence level above the cohort’s median of eight. When considering the midpoint of the scale (5), 86.6% (*n* = 13) of students perceived their confidence as being above the mid-level. Despite this, there was no statistically significant difference in overall perceived confidence of students at the end of T2, regardless of whether initial placement was completed in-house or on outplacement (χ^2^ = 35.000, *p* = 0.328, 95% CI = 0.086–0.267).

Sociodemographic characteristics showed a significant association with professional competencies (Table 2 and Table 3). At the end of T1, there was a significant difference in CT and CL between students with a previous tertiary degree and those without, *p* < 0.05. Students with a previous tertiary degree reported a higher median level of confidence in CT (*n* = 26, 38.2%) and CL (*n* = 25, 36.8%) than students without a tertiary degree (*n* = 13, 19.1%; *n* = 12, 17.6%, respectively).

At the end of T2, a significant difference was shown in SCK and HP (Table 3) between students with previous tertiary degrees and those without (*p* < 0.05). Students with a previous tertiary degree demonstrated a higher median level of confidence in SCK (*n* = 16, 45.7%) and HP (*n* = 16, 45.7%) than students without a tertiary degree (*n* = 3, 8.6%; *n* = 4, 11.4%, respectively). Moreover, there was a statistically significant difference in SCK and CT between different age groups, *p* > 0.05. In SCK and CT, those aged 26–35 had the highest number of students reporting more than the median level of confidence (*n* = 13, 37.1%; *n* = 14, 40.0%, respectively).

The level of perceived confidence in professional competencies among FYDSs had no significant association with the location of placement training received in T1 (*p* > 0.05). Similarly, at the end of T2, the results showed no statistical significance in the level of perceived confidence, irrespective of placement sequence (*p* > 0.05) (Table 4).

### 3.2. Survey 2

Survey 2 consisted of qualitative and quantitative data collection and analysis to assess final-year dental students’ experiences during their clinical placements. With the qualitative data, thematic analysis was employed to identify recurring patterns in their responses. Five key themes were identified, spanning supervisor support and feedback, clinical exposure and patient interaction, learning and skill development, the need for more opportunities, and challenges requiring improvement. The majority of students considered constructive feedback and guidance from their supervisors to be a significant positive contributor to their learning and confidence in clinical practice. Clinical exposure and patient interaction were frequently emphasised as being valuable. The students indicated an appreciation of the opportunity to manage real cases and develop practical skills. Moreover, learning and skill development were denoted as an important outcome, especially in treatment planning and surgical and case management scenarios. However, some students identified increased exposure to endodontics and fixed prosthodontics as an unmet need. Patient availability and logistical constraints were identified as areas requiring improvement.

Content analysis was performed by classifying the responses into six meaningful groups spanning positive feedback on placement (22 responses), challenges faced during placement (4 responses), supervisor feedback and assistance (27 responses), clinical skills and learning, patient interaction and case exposure (25 responses), and suggestions for improvement, as well as varied responses. Varied responses were the suggestions that did not all fall clearly into a single theme.

Most of the students provided positive feedback, highlighting the supportive learning environment and structure of outplacement training programmes. The role of supervisors in facilitating learning, treatment planning, and provision of constructive feedback was appreciated. Students also reported hands-on experience in managing challenging clinical procedures such as surgical extractions and complex restorative procedures as positive experiences. Interactions with patients from diverse backgrounds were also reported as positive aspects in clinical skill acquisition. Only a few students provided negative feedback on limited patient availability and inefficiencies in scheduling appointments, while the suggestions for improvement provided included increasing the patient flow, extending placement duration, and optimising clinical appointment schedules.

A sentiment analysis was conducted to assess emotional tone of the students’ responses. The responses were classified as positive, neutral, or negative. The results indicated that the majority of the FYDSs (91%) expressed positive sentiment. They referred to supportive supervisors, extensive clinical exposure, and well-organised placements as key strengths. A small portion of responses (6%) were neutral. Only 3% of responses exhibited negative sentiment and their primary concerns were related to patient availability and procedural limitations. The overwhelmingly positive sentiment suggests that students found their clinical placements to be a beneficial learning experience.

The analysis of six key items with numerical values in the Likert scale reflected a high level of satisfaction with the clinical placement experience, with a mean of 8.6 (SD = 1.79) and a median of 9 (Table 5). Thus, the results revealed a highly positive student experience, expressed with supervisor support, feedback, and the overall learning environment. Interestingly, the lowest score (mean 7.9) was reported for confidence to work in industry.

All items showed left skewedness, indicating most of the students had positive experiences or high confidence, while only a few students reported lower scores.

The higher kurtosis markedly seen in valuing the placement and the confidence to work obtained from the placement showed that scores were at the higher end of the scale, with a few students giving notably lower ratings.

## 4. Discussion

The dentistry programme referred to in this project was revised in 2019 and continued to evolve and develop in the following years, with evidence-based revision of teaching methods and content to conform to the ADC graduate competencies. The programme received re-accreditation from the ADC in 2022. This study provided insights into FYDSs’ perceived level of confidence in achieving competencies expected by the ADC [5], necessary for safe and effective dental practice.

FYDSs demonstrated high levels of perceived confidence in all the competencies within CL, HP, CT, SCK and PCC. Notably, the median level of confidence was approximately 8 on the 10-point linear numeric scale for each competency. This was consistent with the overall confidence at the end of T1 and T2, irrespective of in-house or outplacement training. These findings provide a positive perspective on current education processes in preparing the future dental workforce. Contemporary research reports high levels of perceived confidence among dental students in specific specialties such as periodontology, restorative, orthodontics, and endodontics [18]. In contrast, the present study evaluated broader, integrated competencies.

The current study developed the questionnaire with reference to the 2022 ADC professional competency document for assessing FYDS’s competencies. Sun et al. developed another valid and reliable scale to assess transferable skills expected from dental graduates using a sample of students from the same university [19]. This scale included factors such as critical thinking, health promotion, communication and leadership, and professionalism and teamwork, which closely mirror the key competencies assessed in the current study. This further verifies the robustness of the current study as it aligns with the core competencies expected in the Australian context.

Further studies have assessed the perceived confidence for competencies in dentistry using the American Dental Education Association guidelines [18,20]. To our knowledge, only one study assessed FYDSs’ confidence against the ADC competencies, and only two of the six domains were examined, using the ADC document published in 2010 [12]. This may have under-represented significant findings expected of a new graduate in the excluded domains. Regular evaluation against updated ADC competencies is essential for maintaining relevance and accreditation [3,21]. The current study provides an overview of FYDS’ confidence levels for five ADC domains and demonstrated that outplacement timing did not have an effect on confidence development of students.

Dental education directly influences students’ long-term attitudes toward preventative care in professional practice [22]. It was interesting to note that the HP domain obtained the highest number of students reporting more than the median level of confidence. This may be attributed to the comprehensive focus on preventative care throughout the dental programme, with learning and teaching modules on public oral health, preventative care, and oral health promotion conducted so that students are well-prepared to meet the ADC HP competency. Furthermore, these courses equip students to recognise the role of dentists in improving community oral health and designing and applying strategies to prevent disease at both community and population. However, there are contrasting findings, with 50% of Australia’s ADC-accredited dental programmes not adequately addressing health promotion competencies, often prioritising restorative skills over prevention [23]. This is concerning given the global burden of preventable dental diseases, emphasising the urgent shift from treatment to prevention [23]. Further research is required to determine the effectiveness and impact of health promotion training in dental education and to address the barriers hindering its practice.

Another key finding was that those participants with a previous tertiary degree reported higher confidence levels in CL, HP, and SCK when compared to those with only secondary education qualifications. This aligns with landmark research in metacognitive studies, which indicates that higher education enhances critical thinking as it requires prolonged engagement with complex concepts and encourages reflective practices [24,25]. Similarly, multiple studies report that students with previous higher education demonstrate better academic performance and higher levels of maturity, motivation, and commitment [26,27,28]. These traits may contribute to their enhanced communication, leadership skills, and application of knowledge in clinical settings. This challenges the paradigm of undergraduate entry models and suggests that admissions should shift towards a post-graduate basis [29].

The FYDS in the age range of 26–35 years in this study reported greater confidence in CT and SCK than the other age groups. This aligns with a study that observed higher levels of critical thinking and enhanced decision making ability among older nurses, which was attributed to their age-related maturity [30]. Another study among student nurses revealed that critical thinking improves with age, allowing for more advanced reasoning in diverse clinical scenarios [31]. However, other studies report no significant correlation between age and critical thinking, highlighting the need for further research [32,33,34]. Based on the findings of this study, having prior tertiary education and age may influence students’ adaptability, preparedness, and performance in the clinical and academic components of the programme. These aspects need to be thoroughly investigated and considered in the selection process for dentistry training programmes.

Future studies should investigate age-related differences in self-confidence against the recommendations of health regulatory bodies in the countries and explore how curricula can be adjusted to support diverse age groups and optimise learning. This may comprise additional case-based learning, which will provide the opportunity to develop skills in critical thinking, information gathering, treatment planning, and execution of treatments. Furthermore, specific treatment planning sessions which provides individualised guidance in comprehensive management of the students own clinical cases would be beneficial.

This study demonstrated that various demographic factors influence perceived competency levels across different domains. As a result, it is crucial to implement an equitable assessment system within the curriculum to identify students who require additional support in weaker areas. Provision of individualised, equitable preparation will enable these students to transition confidently into their roles as newly qualified dental practitioners. Regular assessment of these professional competencies will ensure the curriculum remains current.

Outplacement clinical experience is an integral aspect of dental clinical training. Students collaborate and gain greater exposure to clinical procedures on outplacement, which provides valuable opportunities to enhance their knowledge and work in ‘real-world’ situations before graduating [35]. The findings of the current study showed the majority of the final-year dental students had positive experiences and also emphasised the significance of supervisor engagement, clinical exposure, and skill development in shaping students’ positive experiences during their outplacement training. Improvements in competence and confidence from outplacement have been studied among FYDSs previously and showed similar results [13,20]. Another study assessing the impact of outplacement duration reported that students with 21 weeks of outplacement experience had significantly improved competency in patient management and communication skills than those on a 3-week outplacement [20]. The present study found both in-house and outplacement training (each with a 17-week duration) equally influenced the development of competency levels of the students. Furthermore, the timing of outplacement training did not show a significant difference in perceived confidence. This could be attributed to the low sample size of the data collected, which hindered the relationship from being defined. Increasing the sample size might provide more robust results and insights into underlying patterns.

The limitations of this study include the self-reported nature of the responses. Self- reporting studies are inherently influenced by an individual’s feelings at the time the questionnaire is completed. There is a possibility that students may have overstated their confidence due to social desirability bias. Moreover, as the responses were obtained on a linear numeric scale, some students may have agreed with the statements despite their actual perception, leading to acquiescence and central tendency biases. However, given the resources and the time frame, self-reporting was the most practical way to collect data for this study. Distributing the survey via email potentially impacted the response rate, leading to a notable discrepancy between surveys T1 and T2. The lower response in survey T2 could be due to increased stress and limited availability during the final months of dental training.

SRP is a heterogenous domain consisting of social, cultural, behavioural, and economic factors which impact individual and community health, the provision of culturally safe care to diverse groups and populations, recognising barriers to accessing care, and responding to the distinct needs of those at increased risk of poor oral health. Due to this complexity, the SRP domain was not assessed in the current study and requires investigation at a future date, together with the impact of the psychosocial background of the FYDSs. The SRP domain was excluded due to the large number of survey questions, which can lengthen the questionnaire, causing a negative impact on the response rate. Furthermore, assessing the perceived confidence of FYDSs in the SRP domain before they complete the entire dentistry training may not be useful.

The strengths of this research include its insight into areas of the dental curriculum that require enhancement, as well as the perceived confidence levels of FYDSs upon graduation within the Australian context. This study investigated whether the timing of placement affects confidence levels, a factor that has been given little attention in the literature. Moreover, the present study adds insight to the current literature by investigating the perceived confidence of FYDSs against the most recent version of the ADC competencies expected of newly qualified dental professionals. Prior to the generalisation of this study’s findings for Australia, a collaborative multi-centre study with other dental programmes is required.

## 5. Conclusions

This study’s primary goal was to determine the overall perceived confidence of FYDS against the 2022 ADC competencies. Overall, participants reported a satisfactory level of perceived confidence. Both in-house and outplacement clinical training provided equal levels of perceived competency among FYDSs. There was no significant difference between placement sequence and resulting confidence.

There was a statistically significant association between previous tertiary qualifications and the domains ‘scientific and clinical knowledge’, ‘health promotion’, ‘critical thinking’ and ‘communication and leadership’. The findings will assist clinical educators align curricula and clinical training to better support FYDSs in achieving perceived confidence on graduation.

## Figures and Tables

**Table 1:** The sociodemographic characteristics of final-year dental students in T1 and T2.

Characteristic	No of Students in T1	(%)	No of Students in T2	(%)
**Age**				
18–25 years	32	47.11	12	34.3
26–35 years	29	42.6	18	51.4
36–45 years	7	10.3	3	8.6
Above 45	0	0	2	5.7
Total	68	100.0	35	100.0
**Gender**				
Male	33	48.5	12	34.3
Female	34	50.0	21	60.0
Non-binary	1	1.5	2	5.7
Total	68	100.0	35	100.0
**Student status**				
Domestic	47	69.1	25	71.4
International	21	30.9	10	28.6
Total	68	100.0	35	100.0
**Previous tertiary degree**				
Completed	37	54.4	23	65.7
Not completed	31	45.6	12	34.3
Total	68	100.0	35	100.0
**Placement**				
In-house (university)	26	38.2	20	57.1
Outplacement	42	61.8	15	42.9
Total	68	100.0	35	100.0

**Table 2:** Final-year dental students’ perceived confidence in professional competencies and their sociodemographic characteristics at the end of T1 (*n* = 68).

Variables		Less than Median Level of Confidence N (%)	More than Median Level of Confidence N (%)	Fisher’s Exact Test Value	*p-*Value
CT	Completed a previous degree	11 (16.2)	26 (38.2)	5.537	0.019
	Did not complete a previous degree	18 (25.6)	13 (19.1)		
	Total	29 (42.6)	39 (57.4)		
CL	Completed a previous degree	12 (17.6)	25 (36.8)	5.663	0.017
	Did not complete a previous degree	19 (27.9)	12 (17.6)		
	Total	31 (45.6)	37 (54.4)		

**Table 3:** Final-year dental students’ perceived confidence in professional competencies and their sociodemographic characteristics at the end of T2 (n = 35)**.**

Variables		<Median Confidence	>Median Confidence	Fisher’s Exact Test Value	*p-*Value
		N (%)	N (%)		
HP	Completed a	7 (20.0)	16 (45.7)	4.227	0.040
	previous degree				
	Did not complete a previous degree	8 (22.9)	4 (11.4)		
	Total	15 (42.9)	20 (57.1)		
SCK	Completed a previous degree	7(20.0)	16 (45.7)	6.311	0.012
	Did not complete a previous degree	9 (25.7)	3 (8.6)		
	Total	16 (45.7)	19 (54.3)		
SCK	18–25 years	9 (25.7)	3 (8.6)	6.547	0.040
	26–35 years	5 (14.3)	13 (37.1)		
	36 years and above	2 (5.7)	3 (8.6)		
	Total	16 (45.7)	19 (54.3)		
CT	18–25 years	9 (25.7)	3 (8.6)	8.486	0.014
	26–35 years	4 (11.4)	14 (40.0)		
	36 years and above	3 (8.6)	2 (5.7)		
	Total	16 (45.7)	19 (54.3)		

**Table 4:** Final-year dental students’ perceived confidence in professional competencies by T1 and T2 Placement (*n* = 68 for T1; *n* = 35 for T2)**.**

Variable	Group	<Median Confidence	>Median Confidence	Total N	Fisher’s Exact Test Value
PCC	T1 outplacement	20 (47.6%)	22 (52.4%)	68	0.036
PCC	T1 in-house	13 (50.0%)	13 (50.0%)		
PCC	Total	33 (48.5%)	35 (51.5%)		
SCK	T1 outplacement	9 (45.2%)	23 (54.8%)	68	0.005
SCK	T1 in-house	12 (46.2%)	14 (53.8%)		
SCK	Total	31 (45.6%)	37 (54.4%)		
HP	T1 outplacement	16 (51.6%)	26 (61.9%)	68	2.486
HP	T1 in-house	15 (57.7%)	11 (42.3%)		
HP	Total	31 (45.6%)	37 (54.4%)		
CT	T1 outplacement	18 (42.9%)	24 (57.1%)	68	0.002
CT	T1 in-house	11 (42.3%)	15 (57.7%)		
CT	Total	29 (42.6%)	39 (57.4%)		
CL	T1 outplacement	19 (45.2%)	23 (54.8%)	68	0.005
CL	T1 in-house	12 (46.2%)	14 (53.8%)		
CL	Total	31 (45.6%)	37 (54.4%)		
Overall confidence	T1 outplacement	19 (45.2%)	23 (54.8%)	68	0.005
Overall confidence	T1 in-house	12 (46.2%)	14 (53.8%)	68	
PCC	T2 in-house	10 (50.0%)	10 (50.0%)	35	0.038
PCC	T2 outplacement	7 (46.7%)	8 (53.3%)		
PCC	Total	17 (48.6%)	18 (51.4%)		
SCK	T2 in-house	9 (45.0%)	11 (55.0%)	35	0.01
SCK	T2 outplacement	7 (46.7%)	8 (53.3%)		
SCK	Total	16 (45.7%)	19 (54.3%)		
HP	T2 in-house	6 (30.0%)	14 (70.0%)	35	3.15
HP	T2 outplacement	9 (60.0%)	6 (40.0%)		
HP	Total	15 (42.9%)	20 (57.1%)		
CT	T2 in-house	10 (50.0%)	10 (50.0%)	35	0.345
CT	T2 outplacement	6 (40.0%)	9 (60.0%)		
CT	Total	16 (45.7%)	19 (54.3%)		
CL	T2 in-house	8 (40.0%)	12 (60.0%)	35	0.614
CL	T2 outplacement	8 (53.7%)	7 (46.7%)		
CL	Total	16 (45.7%)	19 (54.3%)		
Overall confidence	T2 in-house	8 (40.0%)	12 (60.0%)	35	0.00
Overall confidence	T2 outplacement	6 (40.0%)	9 (60.0%)	35	

**Table 5:** Students’ feedback on their outplacement experience**.**

Item	Mean	Median	Standard Deviation	Skewness	Kurtosis	Minimum	Maximum
During my placement I was able to perform the required skills and learnt new skills	8.4	8.0	1.45	−0.47	−0.40	5.0	10.0
During my placement I was provided feedback on my clinical performance when requested	8.9	10.0	1.43	−1.25	0.47	5.0	10.0
During my placement I received constructive feedback from all clinical supervisors	8.5	9.0	1.66	−1.02	0.23	4.0	10.0
During my placement I felt comfortable asking for support	9.2	10.0	1.23	−1.30	0.23	6.0	10.0
How would you rate the value of your placement overall	8.6	9.0	1.79	−1.65	2.28	3.0	10.0
Following the placement, I feel confident to work in industry	7.9	8.0	1.63	−1.37	2.68	3.0	10.0

*n* = 103 (T1 and T2 responses).

## Data Availability

The raw data supporting the conclusions of this article will be made available by the authors on request.

## Abbreviations

The following abbreviations are used in this manuscript:

ADC	Australian Dental Council
CL	Communication and Leadership
CT	Critical Thinking
FYDSs	Final-Year Dental Students
HP	Health Promotion
PCC	·Person-centred Care
SCK	Scientific and Clinical knowledge
SD	Standard Deviation
SRP	Social Responsibility and Professionalism
T1	Trimester 1
T2	Trimester 2

## Appendix A

Survey 1 Questions

**Section A: Demographics**

1. What is your gender?
  ○ Female
  ○ Male
  ○ Non-binary
  ○ Prefer not to say
2. What is your age?
  ○ 18–25 years
  ○ 26–35 years
  ○ 36–45 years
  ○ 46–55 years
  ○ Above 55 years
3. What is your student status?
  ○ Domestic student
  ○ International student
4. Have you completed a tertiary level degree before commencing the Bachelor of Dental Health Science?
  ○ Yes
  ○ No
  ○ Unsure
  ○ Prefer not to say
5. Have you completed the final year in house training at Griffith University
  ○ Yes
  ○ No
6. Have you completed the final year out placement training
  ○ Yes
  ○ No

**Section B: Your Confidence in the ADC Dental Competencies**

Please answer all the questions regarding your confidence in these dental competencies. Survey questions are adapted from Australian Dental Council Professional Competencies. A score of 1 indicates ‘no confidence’, 5 indicates confident, and 10 indicates ‘very confident’.

**Statement**	**1**	**2**	**3**	**4**	**5**	**6**	**7**	**8**	**9**	**10**
**1. Communication and leadership**										
I can engage respectfully with the person receiving care, their families, carers, and communities in relation to oral health										
I can present information in a manner that enables the person to understand the care and treatment options available, the risks and benefits, and to be involved in decision making about their care										
I can engage in interprofessional collaborative practice to provide person-centred care										
I can recognise, assess, and respond to domestic and family violence risk, prioritise safety, provide information, and refer as required										
I can engage in mentor/mentee activities and leadership within a healthcare team										
I can maintain one’s own health and wellbeing and support the health and wellbeing of colleagues and team member										
I can utilise digital technologies and informatics to manage health information and inform person-centred care										
I can apply the principles of open disclosure in incident management, review adverse events, and implement changes to reduce the risk of reoccurrence										
I can identify opportunities for improvement in care delivery and advocate for improved oral health outcomes, including for groups or populations at increased risk of harm or poor oral health										
**2. Critical thinking**										
I can locate, critically appraise and evaluate evidence in a scientific manner to support and deliver oral health care										
I can apply clinical reasoning and judgement in a reflective practice approach to oral health care										
I can demonstrate an understanding of research processes and the role of research in advancing knowledge and clinical practice										
**3. Health promotion**										
I can understand the social determinants of health, risk factors and behaviours that influence health										
I can understand the connection between health promotion and health policy development										
I can apply the theories and principles of health promotion to improve oral and general health										
I can design, implement and evaluate evidence-based health promotion strategies and programs										
**4. Scientific and clinical knowledge**										
I can apply the social, cultural, biological, biomedical, physical and behavioural sciences in relation to oral health care provision and disease prevention										
I can apply the theories and principles of population oral health										
I can apply the scientific principles of infection prevention and control										
I can understand the scientific basis, limitations, risks of dental materials and demonstrate their use										
I can apply the principles of risk management and quality improvement										
I can apply the principles of pharmacology, understanding the limitations and risks of using therapeutic agents, including polypharmacy and overuse, and the implication of the Prescribing Competencies Framework on dental practice										
I can understand the scientific basis, risks, and demonstrate the safe use of ionising radiation										
**5. Person centred care**										
**5.1 clinical information gathering**										
I can obtain and record a relevant history of the individual’s medical, social, dietary, and oral health status										
I can perform an examination for health, disease and abnormalities of the dentition, mouth and associated structures										
I can select necessary clinical, pathology and other diagnostic procedures and interpret results										
I can maintain accurate, consistent, legible and contemporaneous records of patient management and protect patient privacy										
I can evaluate individual patient risk factors for oral disease										
I can request and/or take radiographs relevant to dental practice										
**5.2 diagnosis and management planning**										
I can recognise health as it relates to the individual, taking into consideration medical, social and cultural contexts										
I can diagnose disease or abnormalities of the dentition, mouth and associated structures and identify conditions which require management										
I can determine the impact of risk factors, systemic disease and medications on oral health and treatment planning										
I can determine when and how to refer to the appropriate health and or care professional										
I can obtain and record informed consent and financial consent for treatment										
I can formulate and record a comprehensive person centred, evidence-based oral health treatment plan										
**5.3 clinical treatment and evaluation**										
I can apply the principles of disease and trauma prevention and early intervention in the management of the dentition, mouth and associated structures										
I can ensure the principles of supported decision making and positive behaviour support are incorporated into the provision of person-centred care										
I can evaluate and monitor the progress of treatment and oral health outcomes										
I can manage dental emergencies										
I can manage medical emergencies										
I can manage diseases, pathology, and conditions associated with the dentition, orofacial complex, and associated structures										
I can administer, apply and/or prescribe medicines										
I can manage dental caries										
I can manage diseases and conditions of the periodontium and supporting tissues of the teeth or their replacements										
I can manage skeletal and dental occlusal discrepancies										
I can manage the removal of teeth and oral surgical procedures										
I can manage diseases and conditions of pulpal and periapical tissues										
I can manage the loss of tooth structure by restoring the dentition with direct and indirect restorations										
I can utilise removable prostheses to rehabilitate, restore appearance and function, prevent injury, and stabilise the occlusion										
I can utilise fixed prostheses to rehabilitate, restore appearance and function and stabilise the occlusion										
I can manage the individual’s anxiety and pain related to the dentition, mouth and associated structures										

## Appendix B

Survey 2 Questions

Please answer the questions/statements regarding your outplacement training A score of 1 indicates ‘not satisfied’ and 10 indicates ‘very satisfied’.

1. Start date
2. Completion date
3. What was your placement site?
4. How was the accommodation?
5. How did your supervisor assist your learning during your placement?
6. What worked well and what did you like about your placement?
7. What improvements could be made to the placement to add value to your degree and outcome?
8. What aspects of the placement did you find most worthwhile?
9. What aspects of the placement could be improved?
10. Reflecting back on the placement is there anything else you would like to comment on?

	**Statement**	**1**	**2**	**3**	**4**	**5**	**6**	**7**	**8**	**9**	**10**
1.	During my placement I was able to perform the required skills and learnt new skills.										
2.	During my placement I was provided feedback on my clinical performance when I requested it.										
3.	During my placement I received constructive feedback on my work from all clinical supervisors.										
4.	During my placement felt comfortable asking for support.										
5.	How would you rate the value of your placement overall?										
6.	Following the placement, I feel confident to work in industry.										
